# Supplementary material for: Access to Maternity Protection and Potential Implications for Breastfeeding Practices of Domestic Workers in the Western Cape of South Africa
Source: Int J Environ Res Public Health. 2023 Feb 4;20(4):2796. doi: 10.3390/ijerph20042796 (PMC9956374; doi:10.3390/ijerph20042796)
Supplement: Supplementary file 1 [file ijerph-20-02796-s001.zip › ijerph-2120222-supplementary.pdf]

International Journal of
Environmental Research and Public Health

MDPI

Supplementary Material S1: Questions on Maternity Protection to Add to the Annual SweepSouth Survey on Pay and Working Conditions for Domestic Work in South Africa (2021)

The following questions relate to working conditions for domestic workers who are pregnant or breastfeeding, or following the birth of a baby. Even if you have not been pregnant or had a baby while working as a domestic worker, please answer these questions to indicate what you think would happen in the following situations.

The next set of questions is being asked as part of data collection for research being conducted by Katie Pereira-Kotze as part of her PhD in Public Health through the University of the Western Cape. The overall aim of this research is to examine current maternity-health and economic-protection benefits available and accessible to non-standard employees and domestic workers in the Cape Metropole and improve understanding of potential implications for breastfeeding practices. The information collected from these questions in the survey will only be shared with the researchers and they will use it to describe the maternity benefits that domestic workers are currently able to access (or not access). The information received will be used to provide recommendations on how maternity protection for domestic workers can be improved.

Do you want to answer questions on maternity protection for domestic workers? Yes/No.

If yes, please complete the next three questions.

1. **Do you think that a domestic worker who is pregnant at the moment is allowed to receive any of the following benefits?** *(Please click on any answers you think would apply.)*

a) Paid time off work during her pregnancy to attend pregnancy (antenatal) clinic visits.
b) Unpaid time off work during her pregnancy to attend pregnancy (antenatal) clinic visits.
c) Have her employer make changes to the tasks she has to carry out during her work so as not to cause any harm to her or her baby during her pregnancy (for example, not have to lift heavy objects or bend over towards the end of her pregnancy).
d) She should not be allowed to lose her job just because she is pregnant or will be having a baby.
e) She should not be discriminated against because she is pregnant or will be having a baby (for example, her pay should not be reduced because she is pregnant; if starting with a new employer, the employer should not state that she cannot fall pregnant).
f) Do not know.

2. **If you, or a domestic worker in a similar position to you, were to fall pregnant and have a baby, which maternity benefits do you think you or she would be able to receive?** *(Please choose one answer.)*

a) No maternity leave, or less than 6 weeks leave (after the baby is born).
b) Some maternity leave (more than 6 weeks and less than 4 months of leave after the baby is born).
c) Four months of unpaid maternity leave.
d) Four months of partially paid maternity leave.

e) Four months of maternity leave and can claim from the UIF.
f) Four months of full paid maternity leave (organised by the employer).
g) Do not know what is allowed.

3. Do you think that when a domestic worker returns to work after maternity leave, she is allowed to: *(Please click on any answers you think would apply.)*
a) Take paid time off work to attend baby (postnatal) clinic visits?
b) Take unpaid time off work to attend baby (postnatal) clinic visits?
c) Take daily breastfeeding breaks (at least one break during the working day to either express breast milk or breastfeed the baby)?
d) Bring her baby to work with her?
e) None of the above.
f) Do not know.

4. If you have been pregnant or had a baby in the last 3 years, would you be interested in taking part in a follow-up interview with us to discuss in more detail the maternity benefits a pregnant woman or a mother who has just had a baby is able to receive, as well as your breastfeeding experience?
    a) Yes
    b) No

Supplementary Material S2: Question guide for individual in-depth interviews with domestic workers to determine factors influencing the accessibility of maternity-protection benefits and infant-feeding practices upon return to work.

COVID-screening protocol and safety measures

- Meet participants outside admin entrance.
- Participants and researcher to be screened every day by SAMRC before entering building (temperature, COVID symptoms) and issued a sticker that is to be visible throughout the day.
- Have the window and door open at all times (before, during, and after interviews).
- Ensure time (at least 10–15 min) after one participant leaves and the next participant arrives.
- Have sufficient masks available for each participant and participants to always wear a mask, from entry to the building and at all times when in the building (including during the interview).
- Participants to wash their hands before entering the interview room.
- Hand sanitiser available for each participant to use upon entering the interview room.
- Wipe down surface of desk with paper towel and disinfectant between each participant.
- Interviewer and interviewee to sit 1.5 metres apart.

COVID-screening questions

Call participants the day before the interview to ask the following questions before coming for the interview:

- Do you currently have COVID-19? (Have you tested positive for COVID-19 in the past 7 days?)
- Do you live with someone who has either tested positive for COVID-19 or had symptoms of COVID-19 in the past 14 days?
- Are you or any member of your household waiting for a COVID-19 test result?
- In the last 10 days, have you had contact with someone with a suspected or confirmed diagnosis of COVID-19?
- Have you had fever in the last 14 days?
- Have you had a cough in the last 14 days?
- Have you had difficulty breathing in the last 14 days?

References:
https://www.health.gov.za/covid19/assets/downloads/faq/Screening%20and%20testing.pdf
https://www.airports.co.za/Documents/Domestic%20Screening%20%20Questionaire.pdf

Before starting interview: mask, sanitiser, participant information sheet, informed consent form (signed)

Question guide for IDIs with domestic workers

1. When did you have your baby?
2. How many people were you working for when you had the baby?
3. When did you tell your employers that you were pregnant?
4. What was their response?
5. Were you able to take maternity leave? How long? Paid or unpaid?
6. How did you feed your baby after birth (or while you were on maternity leave)?
7. How did you feed your baby when you went back to work?
8. (Prompt: did you breastfeed, did you give infant formula, did you give both?)
9. Why do you think you fed your baby in that way?

Today, we have are going to be talking about maternity benefits.

10. What do you understand by the term “maternity-protection benefits”?

    [Explain to participants:] Maternity protection is a set of benefits that should be made available to women when they are working and pregnant or breastfeeding. Comprehensive maternity protection includes health protection at the workplace, a minimum period of maternity leave, certain cash and medical benefits, job security, non-discrimination, and daily breastfeeding breaks.

11. Have you heard of these types of benefits before, and if so, which ones are you familiar with? Have you heard of the right to breastfeeding breaks?
12. Can you think of when you were pregnant and breastfeeding and the type of benefits, if any, you received from your employer during that time?
13. If you did receive benefits from your employer, how were you able to use those benefits? (Prompt: For example, if your employer contributed to the UIF, were you able to successfully claim from the UIF?).
14. If you were given maternity-protection benefits, why do you think that your employer made those benefits available to you?
15. If you did not receive any maternity-protection benefits, why do you think that your employer did not make these benefits available to you?
16. What kinds of factors do you think would make it easier for an employer of a domestic worker to provide you with maternity-protection benefits?

17. What do you think would make it practical for a domestic worker to be able to exclusively breastfeed her baby for 6 months, and even continue breastfeeding for longer than that?
18. If you had to go back to work and wanted to carry on breastfeeding, would you express your breastmilk and leave it with whomever was caring for your baby to feed your baby while you were working?
